# Bentall Procedure in a Marfan Syndrome Patient With Reduced Ejection Fraction: A Case Report

**DOI:** 10.7759/cureus.76238

**Published:** 2024-12-23

**Authors:** Ghadeer F Bu Saeed, Manal F Alshammari, Dana S Alamoud Abalkhail, Yasser A Elghoneimy

**Affiliations:** 1 College of Medicine and Surgery, Imam Abdulrahman Bin Faisal University, Dammam, SAU; 2 Department of Cardiac Surgery, King Fahad University Hospital, Dammam, SAU

**Keywords:** aortic aneurysm, aortic regurgitation, bentall procedure, ejection fraction, marfan syndrome

## Abstract

Marfan syndrome (MFS) is an autosomal dominant disorder affecting the connective tissue, often leading to aortic root dilation, aneurysm, and dissection. We report on a 35-year-old Bangladeshi female patient with MFS who presented with chest pain, shortness of breath, and a significant aortic root aneurysm, along with a reduced ejection fraction (EF) of 20%-25%. Imaging confirmed significant aortic dilation, and due to the high risk of mortality, an urgent Bentall procedure was performed. Postoperatively, the patient had an EF of 25% and was doing well at discharge. Timely Bentall procedure in MFS with severe aortic root dilation and reduced EF is critical for improving outcomes and reducing morbidity and mortality.

## Introduction

Marfan syndrome (MFS) is an autosomal dominant disorder affecting the connective tissue. The defective gene in this disorder is the FBN1 gene, located on chromosome 15, which affects the production of fibrillin, a protein found in the connective tissue. It is considered as one of the most frequently encountered inherited conditions with an estimated incidence of one in 3,000-5,000 individuals [1]. Aneurysm formation and aortic regurgitation are major complications associated with MFS, specifically affecting the aortic root and the proximal ascending aorta [2]. In this case, we report a 35-year-old female patient with MFS who presented with severe aortic regurgitation and markedly reduced ejection fraction (EF), highlighting a rare and critical presentation. These complications commonly result in heart failure and require urgent surgical intervention to prevent life-threatening consequences. The prevalence of aortic complications in individuals with MFS is considerable, with many requiring surgical intervention before the age of 40 because of the risk of dissection and rupture [2]. Therefore, the main treatment strategy to effectively reduce the risk of dissection or the development of secondary cardiac dysfunction is the replacement of the dilated aortic root with a valved conduit [3]. The life expectancy of patients with MFS has been significantly enhanced through surgical intervention, particularly by the replacement of the dilated aortic root and ascending aorta [4]. In this report, we present our approach to managing a middle-aged female patient with MFS who presented with a significant aneurysm in the aortic root. We implement a personalized surgical approach based on a thorough evaluation of the patient’s condition before, during, and after the operation. It is important to mention that the surgery that was done on this patient carries a high mortality rate due to reduced EF as it was (20%-25%).

## Case presentation

A 35-year-old Bangladeshi female patient, working as a housemaid, with a known case of MFS presented to the emergency department on December 4 complaining of chest pain, shortness of breath, and palpitation. The pain was retrosternal, radiating to the back aggravated by heavy exertion, and slightly relieved by rest. The patient reported experiencing the same symptoms a year ago, but they were less severe.

Additionally, the patient reported pulsations in the right and left inguinal areas and dysphagia. The patient was diagnosed in Bangladesh with aortic regurgitation more than one year ago with a recommendation for surgical management, but due to social issues, the surgery was not done. On arrival, the patient’s blood pressure was 128/55 mmHg, the heart rate was fluctuating between 93 and 115 bpm, the respiratory rate was 21 breaths/min, and she was afebrile with normal oxygen saturation on room air. Her height was 173 cm, and her weight was 57 kg. Physical examination revealed a water hammer pulse, a harsh systolic murmur audible over the precordium with no clear radiation pattern, Corrigan’s sign, pistol-shot sounds over the femoral artery, and positive Quincke’s pulses.

**Figure 1:**
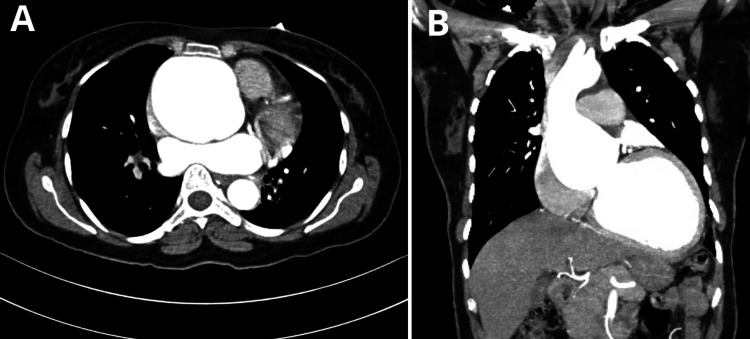
CT angiogram showing (A) axial view with a dilated ascending aorta and (B) coronal view displaying a dilated aortic annulus (2.7 cm) along with a proximal ascending aortic aneurysm (7 cm). CT: computed tomography

Laboratory investigations revealed that amino-terminal pro-brain natriuretic peptide (NT-proBNPT) was markedly increased (8,657.8), indicating severe heart failure. Transthoracic echocardiography (TTE) was done on December 5 and showed a hugely dilated ascending aorta with an aortic root diameter of 7 cm and severe aortic regurgitation. The left ventricle was significantly dilated, and left ventricular function was markedly reduced with an EF of about 20%-25%. Additionally, there was a small intimal flap in the ascending aorta. Computed tomography (CT) angiogram showed a dilated aortic annulus (2.7 cm) (Figure 1A), root (7 cm), and proximal ascending aorta (Figure 1B) with a small dissecting flap at the anterior wall. However, no evidence of complete dissection or false lumen could be identified. Moreover, the left ventricle was shown to be markedly dilated.

**Figure 2:**
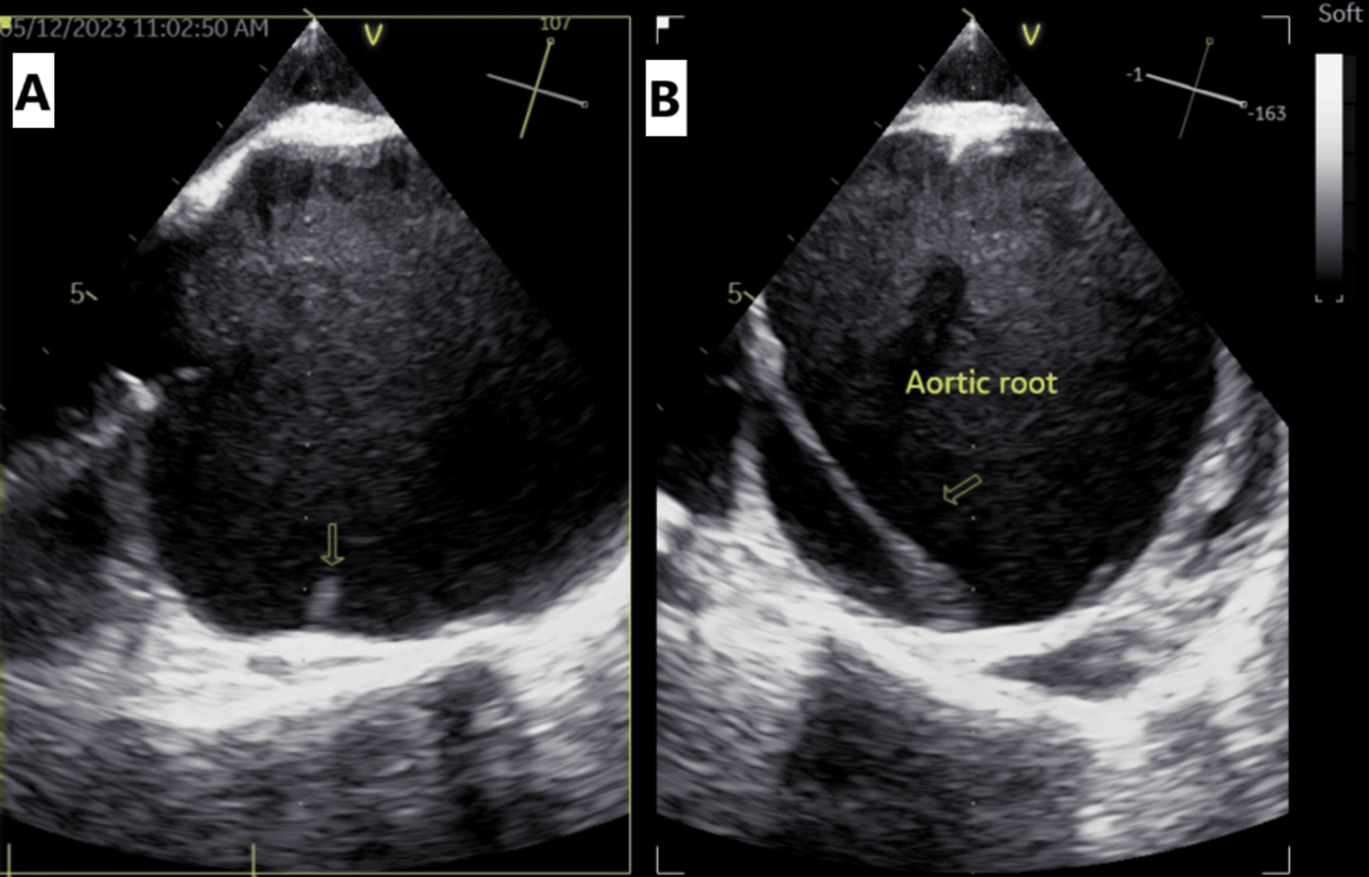
(A,B) TEE showing a dilated aortic root (7.3 cm) with a small intimal flap localized at the aortic root. TEE: transesophageal echocardiography

Transesophageal echocardiography (TEE) revealed similar findings (Figure 2), including severe aortic regurgitation and a dilated aortic root (7.3 cm). Additionally, a small intimal flap was observed near the right coronary ostium, appearing localized to the aortic root. The patient was admitted to the cardiac care unit (CCU).

After a thorough discussion, the team reached a unanimous decision that urgent surgical intervention with the Bentall procedure was deemed necessary. Following informed consent, the patient underwent the procedure on December 11. Intraoperatively, the patient underwent a median sternotomy and was placed on cardiopulmonary bypass. The aorta was cross-clamped, and cold blood cardioplegia was administered antegradely and retrogradely to achieve cardiac arrest. A transverse incision exposed the aortic valve, which was excised. A 27 mm valved conduit was implanted using pledgeted 2-0 Ti-Cron sutures (Medtronic, MN, US). The cardioplegia dose was repeated. Next, the ascending aorta was resected, and the right and left coronary arteries were anastomosed to the tube graft with 5-0 Prolene sutures (Ethicon, Inc., Somerville, US). The distal graft was fashioned and anastomosed to the undersurface of the aortic arch. BioGlue (CryoLife, Inc, Kennesaw, GA, US) was applied to reinforce the suture lines. After de-airing, the cross-clamp was removed, and sinus rhythm was restored after three defibrillation attempts. Protamine was administered, mediastinal and pleural drains were placed, and the chest was closed. The patient tolerated the procedure well and was transferred to the intensive care unit (ICU) in stable condition. The postoperative course was uneventful, the TTE showed EF of 25% with a well-functioning aortic valve, and the patient was discharged 22 days after the surgery in good condition.

## Discussion

Aortic dissection is the most common cause of morbidity and mortality in MFS patients. In our case, the patient presented with chest pain and a dilated ascending aorta, which are both typical of the cardiovascular complications associated with MFS.

Patients with MFS tend to develop aortic dissection at a considerably younger age compared to patients without MFS [4]. The echocardiogram and CT findings confirmed the presence of a dilated aortic root, severe aortic regurgitation, and a small intimal flap, raising concerns about the risk of aortic dissection.

The diagnosis of aortic aneurysm or dissection can be established using various diagnostic modalities such as ultrasound, echocardiography, CT, and magnetic resonance imaging (MRI) [5]. In our patient, TTE and CT angiogram were essential in assessing the severity of the aortic dilation and the risk of dissection, similar to the established diagnostic practices. CT has a crucial role in the diagnosis and risk assessment and in guiding the management of the majority of aneurysms [6]. For our patient, the CT scan was critical in identifying the dilated aortic annulus (2.7 cm), root (7 cm), proximal ascending aorta, and left ventricle.

Once the diagnosis of aortic aneurysm has been confirmed in patients with MFS, it is crucial to implement a thorough management plan aimed at minimizing cardiovascular risks [7]. In this case, our patient had previously been advised to undergo surgical intervention for her aortic regurgitation but had not been able to proceed due to financial constraints. Upon admission, we decided that conservative management was not a viable option due to the significant risk of aortic rupture, necessitating surgical intervention.

Surgical management remains the gold standard treatment approach for aneurysms in MFS [8]. In our patient, the Bentall procedure was chosen due to the severity of her aortic regurgitation and dilation. Although the Bentall procedure is a high-risk surgery, especially in patients with markedly reduced EF like our patient (20%-25%), it was the most appropriate option to prevent imminent aortic rupture. The Bentall procedure, which involves replacing the aortic valve and dilated ascending aorta, is regarded as a long-term solution, with five- and 10-year survival rates of 84% and 75%, respectively [9]. 

Patients with a left ventricular EF (LVEF) below 40% had significantly higher in-hospital mortality (14.0%) compared to those with LVEF between 40% and 49% (5.0%) or above 50% (1.0%), with a p-value of <0.001 [10]. In our case, the patient had an LVEF of 20%-25%, which placed her at a significantly higher risk of mortality and complications during surgery, similar to the higher risk observed in patients with reduced LVEF in the literature.

Additionally, these patients had longer median hospital stays (10.5 days compared to eight and six days, p < 0.001) and extended stays in the ICU (four days compared to two days for both other groups, p < 0.001) [10]. Given the patient's low LVEF, we anticipated the possibility of a prolonged hospital and ICU stay, similar to the patterns noted in the literature.

Meanwhile, valve-sparing aortic root replacement (VSARR) is a preferred surgical option for younger patients who wish to avoid the complications associated with mechanical or biological valve prostheses. This procedure replaces the dilated aortic root while preserving the native aortic valve [9]. In our case, given the patient's dilated aortic root and the condition of the aortic valve, the Bentall procedure with a mechanical valve was deemed more suitable than a valve-sparing approach.

Elective replacement of the aortic root is associated with better outcomes, but in our case, the delay in surgical management due to financial reasons increased the risk of complications, such as the left ventricular dilation and reduced EF we observed. This emphasizes the importance of timely intervention in managing MFS patients with aortic aneurysms.

Since the risk of complications rises markedly around the "hinge point" of 55 mm, surgery is recommended at a smaller dilation threshold (≥50 mm) for the aortic root. Our patient’s aortic root was measured at 7 cm, well above the threshold for surgical intervention, further validating the decision to proceed with the Bentall procedure. Surgery should be considered for aortic root diameters of ≥45 mm in patients with risk factors such as positive family history of dissection, confirmed growth rate >3 mm/year, severe aortic regurgitation, aortic valve intervention necessity, or desire for pregnancy [11].

## Conclusions

This case emphasizes the critical importance of timely surgical intervention in patients with MFS presenting with severe aortic root dilation and reduced EF. Delayed surgical management in such cases significantly increases the risks of morbidity and mortality. The Bentall procedure, when performed promptly, remains an effective and life-saving intervention, as demonstrated by the marked clinical improvement in this patient despite the advanced stage of the disease. This case highlights the essential role of prompt surgical intervention in improving clinical outcomes in patients with MFS and markedly reduced EF.
